# Preventing Adolescent Suicide: Feasibility and Preliminary Outcome Evaluation of a Theatre-Based Gatekeeper Training for Teachers

**DOI:** 10.3390/ijerph21121631

**Published:** 2024-12-07

**Authors:** Chiara Davico, Federica Graziano, Alessandra Rossi Ghiglione, Federico Amianto, Tatiana Begotti, Emanuela Calandri, Giorgia Copetto, Francesca Di Franco, Elena Lonardelli, Daniele Marcotulli, Linda Olcuire, Federica Ricci, Benedetto Vitiello

**Affiliations:** 1 Section of Child and Adolescent Neuropsychiatry, Department of Public Health and Pediatric Sciences, University of Turin, 10126 Turin, Italy; chiara.davico@unito.it (C.D.); francesca.difranco@unito.it (F.D.F.); ele.lonardelli@gmail.com (E.L.); daniele.marcotulli@unito.it (D.M.); linda.olcuire@edu.unito.it (L.O.); federica.ricci@unito.it (F.R.); benedetto.vitiello@unito.it (B.V.); 2 Department of Psychology, University of Turin, 10124 Turin, Italy; tatiana.begotti@unito.it (T.B.); emanuela.calandri@unito.it (E.C.); giorgia.copetto@edu.unito.it (G.C.); 3 Department of Humanities, Social Community Theatre Centre, University of Turin, 10124 Turin, Italy; alessandra.ghiglione@unito.it; 4 Department of Neuroscience, University of Turin, 10126 Turin, Italy; federico.amianto@unito.it

**Keywords:** suicide, prevention, gatekeepers, teachers, performing arts, theatre

## Abstract

Improving teachers’ knowledge and skills in dealing with adolescent suicidality may be important for prevention. We evaluated the feasibility and acceptability of a theatre-based gatekeeper teacher training for adolescent suicide prevention (SPES project). Self-reported changes in knowledge and self-efficacy were also investigated. Based on a quasi-experimental repeated measures design, secondary schools were invited to participate in one of two intervention groups (theatre workshop or attending a theatre performance) or a control group (no intervention). Assessments were conducted prior to the intervention, shortly following it and 3 months later. Attendance and retention were indicators of feasibility. Knowledge and self-efficacy were assessed longitudinally using anonymous questionnaires. Data were analyzed using linear mixed models. 191 teachers (84.3% women; Mage = 46.8, SD = 9.8) participated in the study (63 workshop, 66 performance and 62 control group). Attendance was 92% in the workshop group and 94% in the performance group. The retention rate after three months was 51% in the workshop group and 53% in the performance group. Teachers in both groups reported greater knowledge of adolescent suicide (*p* < 0.001) and higher gatekeeper self-efficacy (*p* < 0.05) than the control group. The SPES project was found to be feasible and acceptable. Both the workshop and the performance viewing may improve teachers’ knowledge and self-efficacy in recognizing signs of suicide risk in adolescents.

## 1. Introduction

Suicide is the second leading cause of death in the 15–24 age group in Italy and other European countries and ranks fourth in the world. Globally, the suicide rate among adolescents (10–19 years old) is 3.77 per 100,000/year [1,2]. The prevalence of suicidal ideation under the age of 22 is estimated to range from 14% to 23%, while the incidence of suicide attempts falls within the range of 5% to 17% [3]. An increase in suicide rate has been reported in some countries over recent years, antecedent to the onset of the COVID-19 pandemic but with worsening in its aftermath [4,5,6,7]. Adolescent suicide prevention must consider the various risk and protective factors, and the settings most relevant to adolescents. Among these settings, school seems to be especially important. Adolescent suicidal behavior is highest during the school year and lowest during the summer months when school is closed [8,9]. These studies suggest that adolescents may experience increased stress and decreased mental health during the school year. Some known risk factors, such as school bullying and academic pressure [10,11] may be related to the observed seasonal patterns of suicidality.

School represents a crucial place for prevention, being the place where students can be educated to take care of their own mental health and acquire protective socio-emotional skills [2,12]. The significant amount of time that adolescents spend in school can be seen as an opportunity to identify students at higher risk for suicide and facilitate appropriate help seeking [13,14,15]. A significant role in suicide prevention can be played by “gatekeepers”, defined as “individuals in a community who have face-to-face contact with a large number of community members as part of their usual routine”. These gatekeepers can be trained to identify individuals at risk and refer them to appropriate psychological support or psychiatric treatment services [16]. This category includes first aid personnel, pharmacists, pediatricians, clergy, healthcare personnel, and teachers.

Teachers, however, often express a lack of confidence in their ability to respond effectively to students displaying signs of suicidality [17,18], feeling that their involvement might exacerbate a crisis [15,19] and generally seeing suicide prevention as a weighty responsibility that requires specific training [20,21]. Gatekeeper trainings can enable teachers to observe any abnormal behavior from students, recognize warning signs of suicide, respond appropriately, and connect at-risk individuals with professional help [22]. Gatekeeper training has been widely implemented in schools and universities, particularly in the U.S.A, where it has been recognized as a promising approach to adolescent suicide prevention [23,24,25,26,27]. However, more supporting evidence for the effectiveness of this approach is needed before wide implementation [28].

A significant limitation of many suicide prevention programs is their dependence on passive training methods, like attending in-person lectures and self-study through online readings and videos [29]. Active learning techniques (e.g., role playing) have shown to be more effective than passive approaches because they promote skill development and empower participants to take charge of their own learning experiences [30,31]. Specifically, the use of performing arts (i.e., participatory theatre, dramatic improvisation, dance, music) has proved to be an innovative and promising methodology in suicide prevention [32]. In particular, theatre fosters open dialogue among participants, reduces the stigma surrounding mental disorders, and improves the expression of emotions and coping skills [33,34,35].

### The Present Study

The SPES project (in Italian, Sostenere e Prevenire Esperienze di Suicidalità: riconoscere il disagio psichico degli adolescenti in alleanza con gli insegnanti; in English: Supporting and Preventing Suicidal Experiences, recognizing adolescents’ mental distress in alliance with teachers), coming from the Latin word “spes” for “hope”, is a gatekeeper training program for secondary school teachers aimed at increasing their knowledge about mental health in adolescence (e.g., depression, non-suicidal self-injury, suicide prevention) and promoting personal and relational competencies (e.g., empathy, communication, ability to collaborate with colleagues, problem-solving abilities). The purpose is therefore to provide teachers with information and tools for managing relationships with young people, recognizing the warning signs of suicide, and increasing collaboration with colleagues and healthcare professionals. The project is based on the Social Community Theatre (SCT) methodology. This is a specific theatrical approach to audience engagement and community development, which motivates participation in a broader process of social and artistic engagement. Ultimately, individuals should come to perceive themselves as integral components of a connected whole. This process of public involvement is fostered by the predominantly nonverbal communicative medium of theatre [35]. The SPES project developed two training formats: a workshop format, based on active and experiential learning, and a format based on the vision of a theatrical performance, both realized according to the SCT approach and combined with a health education intervention on the topic of suicide in adolescence conducted by a healthcare professional. The present study primarily aimed to evaluate the feasibility and acceptability of this training. Teachers’ self-reported outcomes (knowledge about suicide, gatekeeper self-efficacy, teachers’ self-efficacy in collaborating with colleagues, and self-efficacy in managing complex problems) were also examined as exploratory indicators of possible efficacy.

## 2. Materials and Methods

### 2.1. Study Design and Procedure

The study was based on a quasi-experimental repeated measures design with two intervention groups (workshop and performance) and one control group (no intervention) enrolled through a convenience sampling procedure. The team’s goal was to carry out 4 workshops (2 for middle school teachers and 2 for high school teachers), each with about 15–20 teachers. For the performance, the maximum number of expected participants (from both middle and high schools) was about 100. The participant size for both formats was chosen for optimal implementation of activities. Teachers from the same school could participate in only one of the two experimental conditions to avoid contamination. For the characteristics of the intervention, blinding was not achievable.

The research team proposed the workshop to some schools and the performance to others based on existing collaborative contacts and organizational and logistical convenience. The project was presented to school managers with an email followed by a phone call with the request to forward the invitation to the schoolteachers and provide the contact information of those interested. Any subsequent communication was handled by the research team directly with teachers. As for the control group, schools of the same geographical area were contacted through email and phone call to school managers and subsequent direct contacts with interested teachers. The participants received no compensation for taking part in the study. At the end of the study, these teachers were given the opportunity to see the theatrical performance and sign up for a later edition of the project. If they felt uncomfortable completing the questionnaires, they could contact the research staff at any time.

All teachers were assessed at three time points through an anonymous self-report questionnaire administered online. Teachers in the workshop group were assessed before the intervention (T1), the week following the end of the intervention (T2) and the week following the 3-month booster session (T3). Teachers in the performance group were assessed before viewing the performance (T1), the week after the performance (T2) and at 3-month follow-up (T3). Teachers in the control group were assessed at the same time points as the intervention groups. Participants gave written informed consent before completing questionnaires and the research protocol was approved by the Ethics Committee of the University of Turin (protocol number 0576641, 16 November 2022). The study took place in 2023.

### 2.2. Intervention

The intervention was based on the following two formats:

1. Workshop: this format was based on active and experiential learning according to the SCT approach, combined with theoretical-scientific training by experts in child neuropsychiatry. The program consisted of four sessions of three hours each, for four consecutive weeks followed by a “booster” approximately 2 months after the fourth session. Each encounter consisted of a more active segment, followed by a brief health education segment. The sessions were held on the premises of the participating schools and were led by an expert in the SCT approach, while a neuropsychiatrist presented the health education parts (Table 1).
2. Theatrical performance with lecture and debate: teachers participated in a 1 h long theatrical performance, realized according to the SCT approach, and accompanied by a health education intervention on the issue of suicide. The theatre piece was built starting from the stories, musical suggestions, and gestures collected over a long course of theatre workshops conducted with adolescents admitted as inpatients, with the contribution of mental health professionals (i.e., child neuropsychiatrists, nurses, psychologists, pediatricians, and educators) and secondary school teachers. This material was supplemented with readings from studies, the viewing of movies, and works of fiction about the same subject, to enrich the data of workshop experience with scientific research and contemporary imagery on the subject. The life stories told in the play were all based on true events but evoked through theatrical language. The show proposed a dramaturgy of intertwining music videos and recited texts. All the videos in the show were suggested by teenagers as a way to talk about their condition of discomfort. Two professional actors performed during the show. This was followed by a brief lecture about mental health and a Q&A session with the public and a child neuropsychiatrist together with the show’s director. The performance took place in the auditorium of one of the participating schools.

### 2.3. Measures

#### 2.3.1. Demographics

Information was collected about participants’ biological sex, age, level of education, years of professional experience, exposure to suicide, previous experience with suicide prevention and theatre methodology training.

#### 2.3.2. Feasibility

The feasibility of both study implementation (i.e., recruitment, attendance and retention) and study methods (i.e., longitudinal matching and missing data) were evaluated. Recruitment was assessed by the percentage of schools who agreed to participate from among those contacted. Attendance was evaluated by the number of participants in each workshop session. Retention was assessed by the percentage of participants who completed post-intervention (T2) and 3-month follow-up (T3). Differences in attrition were assessed between responders and non-responders in sociodemographic variables (sex, age, type of school, years of experience, exposure to suicide) and baseline scores in the study variables. The matching of questionnaires completed by the same participants at each time point was done through an alphanumeric code filled in by the participants themselves following specific instructions (e.g., first letter of father’s name, mother’s year of birth) to safeguard their anonymity. To assess the method’s feasibility, the percentage of questionnaires matched at post-intervention (T2) and 3-month follow-up (T3) was calculated, as well as the percentage of missing responses at each assessment.

#### 2.3.3. Acceptability

Acceptability was evaluated with a satisfaction questionnaire administered to the teachers of both intervention groups, once immediately after the fourth workshop session and again following the performance. Teachers assessed their overall experience of the training through three closed questions (“How satisfied are you with the training experience?” “Do you think the training was useful for your profession?” “Was the training emotionally challenging?”) each rated on a 4-point Likert scale (from 0 = not at all to 3 = a lot). A fourth closed question asked if participants would recommend the training to a colleague (answer options “yes”, “no”, “don’t know”).

#### 2.3.4. Self-Reported Outcomes

Knowledge about suicide. A 9-item scale was purposefully defined for the study to assess participants’ knowledge about suicide. Questions investigated knowledge of suicide rates among adolescents, main epidemiological data on adolescent suicide, myths and misconceptions about suicide (e.g., “asking whether the person has thought about suicide increases the likelihood of suicide”), risk and protective factors (e.g., “self-harm is a risk factor for suicide”), and main warning signs of suicide (e.g., “social withdrawal can be a warning sign for suicide”). Questions referred to knowledge about suicide useful to the gatekeeper [36,37] and reflect the key contents covered in health education training of both intervention formats. All items were true/false questions. The knowledge score was the sum of the correct responses (1 = correct/0 = incorrect), with higher scores indicating more knowledge (range 0–9).

Gatekeeper self-efficacy. A 7-item scale was specifically adapted from previous studies to evaluate teachers’ perceived ability to recognize warning signs of suicide, communicate with a student with suicidal thoughts, give help, and indicate appropriate health services [18,38]. The questions considered the specificity of clinical referral in the Italian context and the content addressed in the intervention. Each item was scored on a 5-point Likert scale (from 1 = Completely unable to 5 = Completely able; range 7–35) (e.g., “How capable do you feel to communicate appropriately with a student who shows signs of strong psychological distress?”). Reliability in our study was satisfactory (Cronbach’s alpha = 0.85).

Teacher self-efficacy in collaborating with colleagues. A 4-item scale was specifically formulated to evaluate teachers’ perceived ability in collaborating with colleagues to help a student manifesting signs of mental distress. Bandura’s indications for constructing self-efficacy scales were followed [39] (e.g., “How capable do you feel to seek solutions together with colleagues when a student shows signs of psychological distress?”). Each item was scored on a 5-point Likert scale (from 1 = Completely unable to 5 = Completely able; range 4–20). Reliability in our study was satisfactory (Cronbach’s alpha = 0.91).

Self-efficacy in managing complex problems. The scale of perceived self-efficacy in managing complex problems (PSE-MCP) [40] evaluated teacher perceived self-efficacy in coping with problematic life experiences. Teachers were asked to respond with reference to their professional role. Three subscales of the original instrument were selected for the purposes of our study: (1) Emotional maturity: the ability to deal with stressful or unforeseen situations and to have self-control over difficult events (6 items); (2) Relational fluency: the ability to interact with others, to maintain good relationships, to ask for help and to manage conflicts (6 items); (3) Context analysis: the ability to understand connections between different events and situations, to respond to demands from people, and to use a language appropriate to different circumstances (6 items). Each item was scored on a 5-point Likert scale (from 1 = Completely unable to 5 = Completely able). Reliability in our study was satisfactory (Cronbach’s alpha: Emotional maturity = 0.84; Relational fluency = 0.82; Context analysis = 0.90) and comparable with values reported in the Italian validation study [40]. In the present study the total score was used (range 18–90).

### 2.4. Statistical Analysis

Pearson’s chi-squared distribution analyses and univariate analyses of variance were conducted to test for significant differences between the groups at baseline and to investigate differences in attrition between responders and non-responders. A series of linear mixed models (LMMs) was implemented to examine the role of the training (2 intervention groups vs control group) and time (baseline, post-test, and 3-month follow-up), as well as their interaction, as predictors of the examined outcomes. LMMs are more powerful when there is more than one experimental condition and can efficiently handle missing values without deleting the entire case from the analysis [41,42]. The linear mixed models included fixed effects for group (3 conditions), time (3 time points), and the interaction time x group, whereas the intercept was allowed to vary randomly between participants (random effect). The Restricted Maximum Likelihood (REML) estimation method was used. A simple effect analysis was performed to test the effects of time on the different groups. Fit was determined by computing marginal and conditional R^2^ (variance of the fixed effects and variance of both fixed and random effects, respectively) [43]. All the analyses were implemented in Jamovi Version 2.3 [44].

## 3. Results

A total number of 191 teachers were recruited to participate in the study: 63 in the workshop, 66 in the performance, and 62 in the control group (no intervention). The demographic characteristics of the three groups were comparable for sex, age, degree, years of professional experience, exposure to suicide, and prior experience with suicide pre vention and with theatre training. The only difference was in school grade, with more high school teachers in the performance and control groups (Table 2). There were no significant differences between the three groups in any of the study variables at baseline.

### 3.1. Evaluation of Feasibility

**Figure 1:**
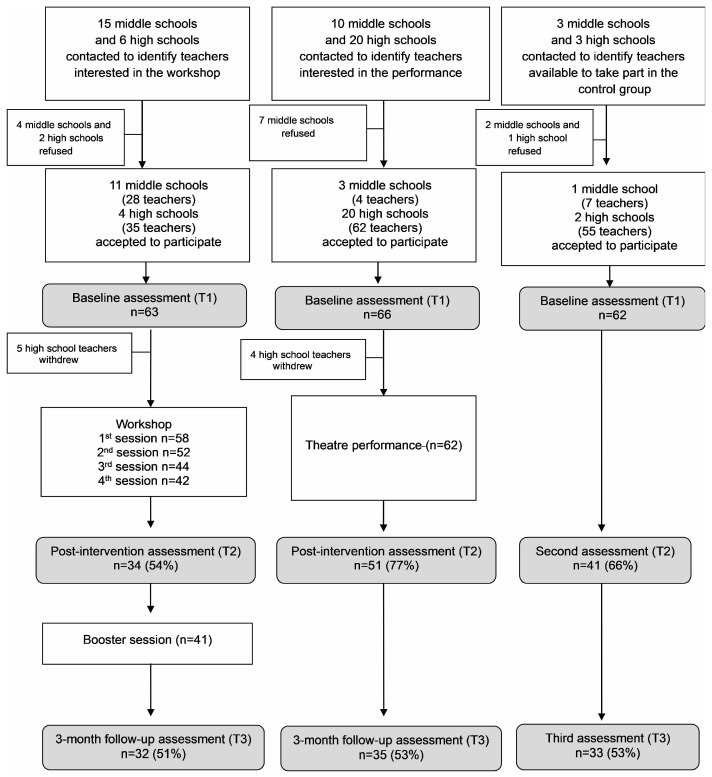
Study flow diagram.

The flow of participants through the SPES project is shown in the CONSORT-adapted flow diagram (Figure 1).

Of 15 middle schools and six high schools invited to the theatrical workshop, 11 (73%) and four (67%), respectively, accepted, including 63 teachers (28 from middle schools and 35 from high schools). Teachers were divided into four groups, each consisting of 14–18 participants. Of 10 middle schools and 20 high schools invited to the theatre performance, three (30%) and 20 (100%), respectively, agreed to participate, including 66 teachers (94% from high schools). Of three middle schools and three high schools in the same geographical area invited to participate as the control group, three accepted (one middle school and two high schools), including 62 teachers.

In the workshop group, five teachers withdrew for personal reasons after the baseline assessment, and six after the first session. The number of teachers present at each session is reported in Figure 1. Of the 63 teachers at baseline, 34 (54%) completed the post-intervention assessment. Forty-one teachers attended the booster sessions and 32 (51% of teachers assessed at baseline) completed the 3-month follow-up assessment. Despite being sent reminders, some teachers did not complete the post-intervention and/or follow-up assessments.

In the performance group, four teachers withdrew after the baseline assessment, 51 (77% of those assessed at baseline) completed the post-intervention assessment, and 35 (53% of those assessed at baseline) completed 3-month follow-up assessment.

In the control group, the second assessment was completed by 41 teachers (66% of those assessed at baseline) and the third assessment was completed by 33 teachers (53% of those assessed at baseline). At post-treatment there were no statistically significant differences between responders and non-responders in sex, age, type of school, years of experience, and baseline scores in the study variables. Responders were more likely to have been exposed to a student who attempted suicide (χ^2^ = 10.10, df = 1, *p* < 0.001).

At the 3-month assessment, there were no statistically significant differences between responders and non-responders in sex, age, type of school, years of experience, and baseline scores in the study variables. A statistically significant difference was found only for exposure to suicide: responders were more likely to have been exposed to a student who attempted suicide (χ^2^ = 12.22, df = 1, *p* < 0.001) or committed suicide (χ^2^ = 8.44, df = 1, *p* < 0.005). Retention at post-treatment was higher for teachers in the performance group (χ^2^ = 8.10, *p* < 0.01), whereas retention at follow-up was higher for teachers in the workshop group (χ^2^ = 7.94, *p* < 0.01).

The use of participants’ self-compiled code made it possible to match most of the questionnaires (98% at T2 and 97% at T3). The unmatched questionnaires had small differences in codes and were cross-checked on sociodemographic variables, until 100% of questionnaires were matched. The percentage of missing data at each assessment was <5%. The MCAR (Missing Completely at Random) test was not statistically significant, indicating that the data missing was completely at random.

### 3.2. Evaluation of Acceptability

The satisfaction questionnaire was completed by 38 teachers participating in the workshop and 56 teachers who attended the performance (total n = 94) (Table 3). Teachers were generally satisfied with both training formats. Both formats were evaluated as useful, although emotionally challenging. Most teachers would recommend the program to a colleague. There were no differences between the two groups for any of the indicators considered.

### 3.3. Outcome Evaluation

**Figure 2:**
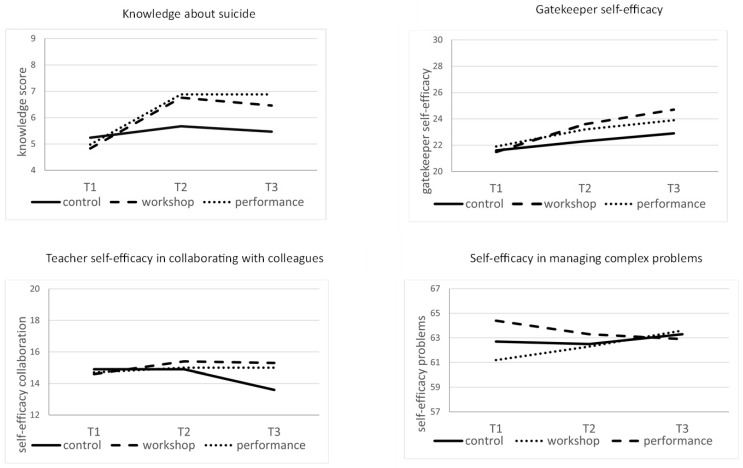
Trajectories of change in the outcomes by condition (workshop, performance and control).

Results showed that there was a significant group × time effect on knowledge about suicide (Table 4). Simple effect analysis showed that there was a significant effect of time for the workshop and the performance but not for the control. Analysis of contrasts between time points (T1, T2, T3) for each group (workshop, performance, control) revealed that knowledge increased from baseline to post-test for both workshop and performance and then remained constant (Table 5 and Figure 2).

A significant group × time effect on gatekeeper self-efficacy was observed. The effect of time was significant for all groups, but stronger for workshop and performance than for the control. Contrasts revealed that the level of gatekeeper self-efficacy increased from baseline to post-test for both the workshop and the performance groups. At follow-up, a slight increase was observed for the workshop, but not for the performance.

Results showed that there was a significant group × time effect on teacher self-efficacy in collaborating with colleagues. The effect of time was significant in the control group and non-significant for workshop and performance: the difference was negative since decreasing scores were observed in the control group. Specifically, contrasts revealed that the level of teacher self-efficacy slightly increased from baseline to post-test for the workshop, whereas a deep decrease in self-efficacy levels was observed among teachers of the control group at 3-month follow-up.

The interaction group × time on the perceived ability to manage complex problems did not reach statistical significance. Results suggested a significant simple effect of time for the workshop, but not for the performance or the control. Contrasts revealed no statistical differences.

## 4. Discussion

This study evaluated the feasibility and acceptability of two theatre-based training formats (a workshop and a theatrical performance), both accompanied by health education to enhance teachers’ knowledge and self-efficacy in recognizing warning signs of suicide risk in adolescents. Using a non-randomized prospective design with a control group (no intervention) for comparison, we found that both intervention formats could be delivered in a feasible and acceptable way and were associated with greater knowledge acquisition and gatekeeper self-efficacy than the control condition. Several considerations emerged from this study that can inform further work on the application of theatrical elements to teacher training for school suicide prevention.

Recruitment was challenging, particularly for middle school teachers, possibly because teachers still consider the issue of suicide to be of little relevance to early adolescents. The two formats presented different results in terms of attendance and retention rate since the commitment required of teachers was very different. Participation in the workshop implied a significant engagement in terms of time and personal resources; thus a smaller attendance rate was expected. However, teachers who agreed to participate in the workshop demonstrated a higher retention rate at 3-month follow-up, likely due to their deep engagement with the experience. As expected, attendance and immediate retention rates were higher for the theatrical performance. Theatrical performance could serve as an effective tool to reach a higher number of recipients. If they are interested in furthering their skill development, participants could be involved in further workshops in a sequential proposal. As for attrition, improvements are needed to increase the response rate to questionnaires by having them filled out immediately at the end of the sessions. We did not find differences in attrition except for exposure to suicide; responders were more likely to have been exposed to a student who attempted or committed suicide, in line with other similar studies [45]. The acceptability of both formats was high. Overall, participants were satisfied with the training, despite the emotional burden, and almost all teachers would recommend the program to a colleague.

The preliminary results regarding the impact on self-reported outcomes are encouraging. Knowledge about suicide increased in both intervention groups. This can be considered a significant result, given that discussions about suicide remain burdened by stigma and prejudice [46]. Despite the critical need to address these themes, very often the public dialogue remains superficial and tends to be simplistic. In a recent study that explored Italian news coverage of adolescent suicide, the authors found that, compared with adult suicide cases, news about cases of teen suicide are given more attention in the newspapers, as shown by a greater number of articles written for each suicide event and more words contained in each article [47]. This heightened media attention may reflect society’s need to comprehend the self-inflicted death of a young person, an act that seems unbelievable and impossible [47]. The WHO has emphasized how improving community awareness and breaking down the taboo about suicide is important for making progress in suicide prevention and has pointed to the need for a multisectoral collaboration [48]. Strong collaboration between health professionals, playwriters, educators, and people coming from the world of theatre allowed us to create a multisectoral intervention program.

Having a good understanding of mental health, suicide prevention and warning signs is not sufficient to enable intervention with students. For this reason, gatekeeper self-efficacy was evaluated. Our preliminary results suggest that both formats can enhance teachers’ abilities in recognizing warning signs of suicide, communicating with a student with suicidal thoughts, giving help and indicating appropriate health services. As previously mentioned, feeling inadequately confident in dealing with suffering pupils is a major barrier to teachers’ ability to take care of their students [15,18,19,20,21].

Another indicator was teacher self-efficacy in collaborating with colleagues; when dealing with a suicidal adolescent, it is crucial that teachers do not feel isolated. In our study, the level of teacher self-efficacy slightly increased from baseline to post-test for the workshop, whereas a deep decrease in self-efficacy levels was observed among teachers of the control group at 3-month follow-up. It is worth noting that the 3-month follow-up was carried out at the end of the school year, a period when teachers are likely to feel more stressed and fatigued after an entire academic year. SPES interventions could represent a protective factor helping teachers maintain their sense of collaboration with colleagues and mitigating the challenges of isolation.

In our study, self-efficacy in managing complex problems did not change significantly over time for either the intervention or control groups, likely due to the general ability to manage complex situations that we explored, rather than a specific assessment of the ability to deal with critical students. In addition, these skills probably more time to be effectively modified.

As for possible differences between the two formats, our preliminary results do not allow us to reach definitive conclusions. Nonetheless, we found that the level of gatekeeper self-efficacy remained higher over time for teachers participating in the workshop. Similarly, results suggest a short-term effect of the workshop on teachers’ self-efficacy in collaborating with colleagues. Although further research is needed to confirm these results, the workshop format could be more useful to enhance these competencies compared to the performance. As previously successfully reported in other suicide prevention programs [30,31], active learning techniques, such as role playing, appear to enhance the learning process. These approaches appear to be superior to passive techniques as they can foster skill acquisition and subsequent behavior, largely through the opportunity to practice and receive constructive feedback on skill use. Role playing enables learners to engage in lifelike scenarios that could potentially induce stress in real-world situations. Considering the great fear of harming that teachers very often report when they think about dealing with a suicidal student, the opportunity to monitor their skill development by practicing learned skills, asking questions and receiving appropriate feedback on their performance through dynamic interactions with trainers seems to be crucial.

Viewing the theatrical performance yielded positive results, especially with reference to knowledge and gatekeeper self-efficacy. This might be related to the interactive and emotionally engaging nature of the intervention. The emotional involvement elicited by the theatrical performance appears to create an opportunity for receptive learning, which in our format is reinforced by the lecture on mental health that immediately follows the performance.

This study has several limitations. First, the allocation to the study groups was not random. The recruitment process, however, was systematic and offered participation to a representative group of schools and teachers. In fact, the baseline characteristics of the three groups were comparable. The sample was mainly female, but this reflects the sex prevalence of the teachers in the community. Middle school teachers were relatively under-represented in the performance condition. A randomized trial involving more participants is needed to evaluate the effectiveness of the SPES project. Second, the follow-up time was limited to three months, whereas future research should monitor the effectiveness of the training in the long term. Third, we did not evaluate actual teacher behaviors following the intervention when faced with students exhibiting mental health challenges. This outcome should be evaluated in future research. Finally, the ultimate effect of the training interventions on adolescents (e.g., suicide attempt rates/consultations at specialized clinics) was not part of the study. This represents a significant limitation, highlighting the need for future studies that validate interventions through testing of their effectiveness with randomized controlled trials [28].

## 5. Conclusions

The SPES project demonstrated good feasibility and acceptability among secondary school teachers. Exploratory outcome assessments indicate that both the workshop and the performance format may improve teachers’ knowledge and self-efficacy in recognizing warning signs of heightened suicide risk among adolescent students. Further research employing randomized controlled methodology is needed to assess the effectiveness of these interventions and their potential contribution to adolescent suicide prevention.

## Figures and Tables

**Table 1:** Description of workshop content.

Session	Experiential Activities	Health Education Topics
1	*Description* Individual training in physical self-perception and emotional awareness Physical identification with adolescents Storytelling: adolescent stories Oral group sharing of life experiences and reflection on the skills learned/discovered	*Description* What is suicide in adolescence: definitions and epidemiological
	*Aims* Recognizing and managing one’s emotions in relation to an adolescent mental health problem Promoting gatekeeper self-efficacy (developing relationship and communication skills) Developing self-efficacy in collaboration with colleagues and other school staff	*Aims* Increasing knowledge about suicidality in adolescence
2	*Description* Individual training in physical self-perception and emotional awareness Individual and couple training on the perception of the other and self in the relationship Role playing: Life scenes of fragile teens—Giving voice to teens’ silent thoughts Group discussion and reflection on the skills learned/discovered	*Description* Risk and protective factors and warning signs for suicide
	*Aims* Recognizing and managing one’s emotions in relation to an adolescent mental health problem Promoting gatekeeper self-efficacy (recognizing possible suicidal intentions) Developing self-efficacy in collaboration with colleagues and other school staff	*Aims* Increasing knowledge about suicidality in adolescence and promoting gatekeeper self-efficacy
3	*Description* Individual training in physical self-perception and emotional awareness Relational training on observing the well-being of the other and listening to the other Role playing: communicating with fragile adolescents Group discussion and reflection on the skills learned/discovered	*Description* How to talk to someone at risk for suicide
	*Aims* Recognizing and managing one’s emotions in relation to an adolescent mental health problem Promoting gatekeeper self-efficacy (developing relationship and communication skills; recognizing possible suicidal intentions)	*Aims* Promoting gatekeeper self-efficacy
4	*Description* Individual training in physical self-perception and emotional awareness Role playing: Life scenes of teachers when there is a very fragile or suicide-prone adolescent in the classroom Group discussion and reflection on the skills learned/discovered Suggested readings and activities to be done before follow-up	*Description* How to become a “gatekeeper”: recognizing and bringing help to students in need, getting in touch with health professionals, what it is and how to cultivate mental health
	*Aims* Recognizing and managing one’s emotions in relation to an adolescent mental health problem Promoting gatekeeper self-efficacy (developing relationship and communication skills; recognizing possible suicidal intentions)	*Aims* Increasing knowledge about health services for adolescents; promoting gatekeeper self-efficacy; promoting teacher awareness of their mental health
5	*Description* Individual training in physical self-perception and emotional awareness Relational training on the experiences that occurred in the period up to follow-up Group discussion and reflection on the skills learned/discovered and experiences with adolescents and colleagues	-
	*Aims* Recognizing and managing one’s emotions in relation to an adolescent mental health problem Developing self-efficacy in collaboration with colleagues and other school staff	-

**Table 2:** Characteristics of participants at baseline.

	Workshop (n %)	Theatrical Performance (n, %)	Control (n, %)	Difference
Biological sex				
Female	54 (85.7)	58 (87.9)	49 (79)	χ^2^ = 2.03, df = 2, *p* = 0.362
Male	9 (14.3)	8 (12.1)	13 (21)	
Age (M, SD)	48.6 (8.9)	45.5 (10.3)	46.4 (10.1)	F_(2,187)_ = 1.63, *p* = 0.199
Education level				
High school	2 (3.3)	1 (1.5)	3 (4.9)	χ^2^ = 1.81, df = 4, *p* = 0.771
Degree	40 (66.7)	42 (64.6)	42 (68.9)	
Post-degree	18 (30)	22 (33.8)	16 (26.2)	
School type				
Middle school	28 (44.4)	4 (6.3)	7 (11.3)	χ^2^ = 33.2, df = 2, *p* < 0.001
High school	35 (55.6)	60 (93.8)	55 (88.7)	
Years of professional experience (M, SD)	16.9 (11.4)	14.6 (11.5)	15.9 (11.6)	F_(2,183)_ = 0.64, *p* = 0.527
Exposure to student suicide				
Suicidal ideation	16 (26.7)	20 (32.3)	13 (22)	χ^2^ = 1.61, df = 2, *p* = 0.448
Suicidal attempt	13 (21.7)	23 (37.1)	22 (37.3)	χ^2^ = 4.44, df = 2, *p* = 0.109
Suicidal act	5 (8.3)	9 (14.5)	2 (3.4)	χ^2^ = 4.67, df = 2, *p* = 0.097
Prior suicide prevention training	2 (3.3)	4 (6.3)	4 (6.5)	χ^2^ = 0.79, df = 2, *p* = 0.675
Prior performing art training	15 (23.8)	14 (21.5)	8 (12.9)	χ^2^ = 2.64, df = 2, *p* = 0.267

**Table 3:** Participants’ responses to the satisfaction questionnaire.

		Workshop (n = 38)	Performance (n = 56)	
How satisfied are you with the training experience?	Quite satisfied	29%	14%	χ^2^ = 3.02, *p* = 0.082
	Very satisfied	71%	86%	
Do you think the training was useful for your profession?	Quite useful	26%	32%	χ^2^ = 0.37, *p* = 0.544
	Very useful	74%	68%	
Was the training emotionally challenging?	A little	3%	2%	χ^2^ = 0.08, *p* = 0.961
	Quite challenging	47%	48%	
	Very challenging	50%	50%	
Would you recommend the training to a colleague?	Yes	95%	98%	χ^2^ = 0.82, *p* = 0.364
	No/don’t know	5%	2%	

**Table 4:** Estimated marginal means in the outcomes by condition and significance of the fixed effects.

	Workshop (M, SE)	Performance (M, SE)	Control (M, SE)	Fixed Effects		
				Group	Time	Group × Time
Knowledge about suicide						
T1	4.83 (0.21)	4.98 (0.20)	5.24 (0.21)	F_(2,189)_ = 4.83 *p* < 0.01	F_(2,234)_ = 56.82 *p* < 0.001	F_(4,234)_ = 7.92 *p* < 0.001
T2	6.76 (0.26)	6.88 (0.22)	5.67 (0.28)			
T3	6.46 (0.27)	6.88 (0.25)	5.47 (0.27)			
Gatekeeper self-efficacy						
T1	21.5 (0.47)	21.9 (0.47)	21.6 (0.48)	F_(2,181)_ = 1.35 *p* = 0.262	F_(2,219)_ = 41.34 *p* < 0.001	F_(4,219)_ = 2.88 *p* < 0.05
T2	23.6 (0.55)	23.2 (0.50)	22.3 (0.53)			
T3	24.7 (0.55)	23.9 (0.54)	22.9 (0.56)			
Self-efficacy colleagues						
T1	14.6 (0.34)	14.7 (0.34)	14.9 (0.35)	F_(2,187)_ = 1.02 *p* = 0.361	F_(2,225)_ = 1.84 *p* = 0.161	F_(4,225)_ = 3.65 *p* < 0.01
T2	15.4 (0.42)	15.0 (0.38)	14.9 (0.41)			
T3	15.3 (0.43)	15.0 (0.42)	13.6 (0.43)			
Self-efficacy complex problems						
T1	61.2 (1.06)	64.4 (1.04)	62.7 (1.08)	F_(2,182)_ = 0.35 *p* = 0.705	F_(2,210)_ = 0.56 *p* = 0.572	F_(4,210)_ = 2.30 *p* = 0.06
T2	62.3 (1.21)	63.3 (1.12)	62.5 (1.19)			
T3	63.6 (1.22)	62.9 (1.18)	63.3 (1.22)			

Note: T1 = baseline; T2 = post-test; T3 = 3-month follow-up; SE = standard error; Knowledge about suicide: R^2^ marginal = 0.21, R^2^ conditional = 0.59, ICC = 0.483; Gatekeeper self-efficacy: R^2^ marginal = 0.07, R^2^ conditional = 0.77, ICC = 0.754; Teacher self-efficacy collaboration colleagues: R^2^ marginal = 0.02, R^2^ conditional = 0.61, ICC = 0.600; Self-efficacy managing complex problems: R^2^ marginal = 0.01, R^2^ conditional = 0.79, ICC = 0.785; ICC = intraclass correlation coefficient.

**Table 5:** Simple effects analysis and contrasts.

	Condition	Simple Effect of Time	*p*	Contrast	t	*p*
Knowledge about suicide	Workshop	F_(2,224)_ = 32.35	<0.001	T1–T2	−7.20	<0.001
				T2–T3	0.99	0.319
	Performance	F_(2,217)_ = 43.78	<0.001	T1–T2	−8.32	<0.001
				T2–T3	0.99	0.99
	Control	F(_2,235)_ = 1.20	0.302	T1–T2	−1.51	0.132
				T2–T3	0.65	0.517
Gatekeeper self-efficacy	Workshop	F_(2,215)_ = 28.93	<0.001	T1–T2	−4.80	<0.001
				T2–T3	−2.35	<0.05
	Performance	F_(2,212)_ = 13.76	<0.001	T1–T2	−3.69	<0.001
				T2–T3	−1.63	0.104
	Control	F_(2,214)_ = 4.28	<0.05	T1–T2	−1.58	0.115
				T2–T3	−1.34	0.181
Self-efficacy colleagues	Workshop	F_(2,222)_ = 2.27	0.069	T1–T2	−2.02	<0.05
				T2–T3	0.15	0.884
	Performance	F_(2,216)_ = 0.71	0.493	T1–T2	−0.98	0.328
				T2–T3	−0.14	0.885
	Control	F_(2,215)_ = 28.93	<0.001	T1–T2	0.10	0.919
				T2–T3	2.82	<0.005
Self-efficacy complex problems	Workshop	F_(2,207)_ = 3.22	<0.05	T1–T2	−1.16	0.247
				T2–T3	−1.26	0.208
	Performance	F_(2,204)_ = 1.58	0.208	T1–T2	1.30	0.194
				T2–T3	0.46	0.649
	Control	F_(2,207)_ = 0.29	0.742	T1–T2	0.15	0.884
				T2–T3	−0.73	0.469

## Data Availability

Data that supports the findings of this study are available from the corresponding author upon reasonable request.
